# Pyrolysis Evaluation of Tennis String Polyurethane and Water-Borne Polyurethane Wastes through Isoconversional Kinetic Analysis

**DOI:** 10.3390/polym14081501

**Published:** 2022-04-07

**Authors:** Haibo Wan, Zhen Huang

**Affiliations:** 1 Department of Physical Education, Tianjin University of Commerce, Tianjin 300134, China; wanhaibo@tjcu.edu.cn; 2 Department of Packaging Engineering, Tianjin University of Commerce, Tianjin 300134, China

**Keywords:** polyurethane, thermogravimetric analysis, non-isothermal pyrolysis, isoconversional kinetic analysis

## Abstract

A detailed kinetic analysis of pyrolysis processes of Tennis string polyurethane (TSPU) waste and waterborne polyurethane (WPU) waste was carried out in the present paper. Non-isothermal pyrolysis characterizations of two wastes were acquired through thermogravimetric determinations under the constant heating rates of 5, 10, 15 and 20 K/min. Experimental results showed that the pyrolysis processes of TSPU and WPU were mainly characteristic of three stages and two stages, respectively. Two pyrolysis performance indices, the devolatilization index (DI) and heat-resistance index (HRI), were used to interpret the heating rate effect on the pyrolysis features and different thermal dependences of TSPU and WPU. Isoconversional kinetic analysis was thoroughly performed with model-free and model-fitting methods. By using Starink, Vyazovkin–Dollimore and Coats–Redfern methods, the activation energies thus obtained were in the range of 103.3~148.3 kJ/mol and 92.5~204.3 kJ/mol, respectively, for TSPU and WPU, over the entire pyrolysis process. Their respective pre-exponential factor lnA values were determined to be within 17.94~33.42 min^−1^ and 16.56~20.82 min^−1^. Thermodynamic parameters in terms of Δ*G*^#^, Δ*H*^#^ and Δ*S*^#^ throughout the entire pyrolysis process were also calculated. Finally, by means of the model-fitting Coats–Redfern method, the most appropriate mechanism functions were ascertained for, respectively, describing multi-stage pyrolysis degradations of TSPU and WPU waste. These results may offer meaningful support for designing any industrial pyrolysis reactor to dispose of polyurethane wastes.

## 1. Introduction

Nowadays, many researchers and entrepreneurs are struggling to solve the overwhelming amount of petroleum-based plastic wastes around the world because plastic refuses has seriously destroyed the global ecological environment and further restrained the rapid development of the social economy and the improvement of people’s living. Of all the plastic wastes, polyurethane (PU) has contributed very much, with its outstanding tensile strength, anti-wearing, anti-abrasion and adhesive force and is thus widely used in the field of shoe-making, sporting goods, furniture products, household appliances, medical supplies and construction materials [1]. PU, characteristic of the urethane bond of −NH−COO−, is known to be composed of the hard segments of isocyanates and the soft segments of polyols with low molecular alcohol as a chain extender [2]. To solve the ever-increasing plastic refuses of significant environmental impact, that cannot be ignored any longer, conventional burning, land-fill and recycling methodologies have still been prevailing even though they potentially involve negative influences on the environment’s protection, land occupation and economic cost [1]. Worldwide, pyrolysis of various plastic wastes has now been regarded as an efficient waste-to-energy technology to convert solid wastes into value-added chemical products.

For pyrolysis investigations, thermogravimetric analysis (TGA) is the most powerful tool. In this way, thermal degradations of various solid wastes can be thoroughly analyzed and kinetic models, thus established eventually, can be used to quantitatively represent pyrolysis processes [3,4,5,6,7,8,9,10,11,12,13,14,15,16,17,18,19,20,21]. Up to now, a number of research scientists have made great efforts over the last few decades to investigate the pyrolysis behaviors of polyurethanes, including rigid foams [3,4,5,6,7], flexible foams [8,9,10,11], tires [12], elastomers [13,14,15,16], waterborne materials [17,18], composites [4,10,15,18,19,20] and wastes [12,20,21]. These works show that for pyrolysis of polyurethane, it is a complex multi-stage degradation process of two-stage [3,4,8,9,10,11,15,16,17] or three-stage [12,16,19,20,21], and sometimes approximated globally as a one-stage process [5,18]. If simplified as a two-stage thermal degradation, stage one may be assumed as the breakdown of the urethane unit to release isocyanates and amines with R-N=C=O groups and yield condensed polyols, while stage two is thought of as the decomposition of the polyols, leaving behind any possible solid residues [8]. On the other hand, if tallying with a three-stage reaction, stage one may be taken as the volatilization of unreacted isocyanate, stage two as the cleavage of the urethane bond and pyrolysis of polyols, and stage three as the isocyanate conversing into anilines and aromatics [21]. Additionally, some scholars have also reported more intricate four-stage processes [6,14] or five-stage processes [5] for pyrolysis of polyurethane. The pyrolysis stage difference seems to be arising from different chemicals used to synthesize polyurethane polymers along with the differences in both molecular weights and synthesis methods.

Apart from studying pyrolysis degradation features, knowledge of pyrolysis kinetics in terms of apparent activation energy (*E_k_*) and pre-exponential factor (*A*) and reaction function *f*(*α*) is of great importance to study as well, resulting from TGA data by means of different kinetic calculation methods. Once correctly ascertained, the kinetic triplet parameters can be attempted to regenerate the pyrolysis degradation data and foresee the pyrolysis features beyond the experimental temperature range as well. Furthermore, the parameter *E_k_* is very helpful to interpret the thermal stability of the materials studied. Therefore, many researchers have made striking efforts to perform kinetic analysis of pyrolysis processes along with experimental determinations [22]. For example, Cui et al. [18] gained *E_k_* = 149.1 kJ/mol, lnA = 22.2 min^−1^ and the reaction order n ≈ 1 for pyrolysis of WPU by assuming a one-stage degradation reaction, and *E_k_* of 130.5~150.7 kJ/mol and lnA of 19.1~22.2 min^−1^ for WPU/silver flake composites. Garrido and Font [8] have found that two consecutive reactions, respectively, with *E_k_* values of 142 and 217.5 kJ/mol and reaction orders of 0.805 and 1.246, can satisfactorily interpret two-stage pyrolysis of flexible polyurethane foam. In the meantime, Rogaume et al. [11] have studied the pyrolysis decomposition mechanism of polyether polyurethane foam and found that for two stages the *E_k_* values of 169.9 and 243.9 kJ/mol are determined along with a reaction order of n = 0.91 and 1.26, respectively. Recently, Tang et al. have investigated pyrolysis of waste polyurethane tires and resolved that three consecutive reactions with three-halves order, first-order, and second-order models can excellently represent the whole process with the aid of the mean *E_k_* of 204.4 kJ/mol. Much earlier, Lefebvre et al. [6] have deeply decoupled pyrolysis of rigid polyurethane foams and unraveled the complex pyrolysis into four continuous stages. They obtained the *E_k_* of 73.7, 174.9, 220.1 and 240.7 kJ/mol, and the pre-exponential factors *A* of 1.36 × 10^5^, 1.13 × 10^14^, 1.94 × 10^14^ and 2.61 × 10^14^ S^−1^, for these stages. Similarly, Rosu et al. [14] also observed at least four pyrolysis stages, that partially overlapped, and thus established four n-order reaction models for an MDI based polyurethane elastomer. However, there are considerable discrepancies among these works if looking through these kinetic parameters, summarily perhaps due to (1) different reactants and methods used to synthesize PU of different molecular weights, (2) different kinetic methods to analyze pyrolysis of PU and (3) different pyrolysis conditions, such as isothermal heating, single- heating rate or multi-heating rate. Therefore, it is necessary to reconsider the kinetic pyrolysis process of PU via widely accepted isoconversional analysis methods based on multiple heating rate TG data, highly suggested by the International Confederation for Thermal Analysis and Calorimetry (ICTAC) Kinetics Committee [22].

In the present work, tennis string polyurethane (TSPU) waste and waterborne polyurethane (WPU) paper-sizing coating waste were considered for the study of their pyrolysis degradations in a nitrogen atmosphere. Up to now, there is no report on the thermal degradation of TSPU fibers and WPU coatings. This work is aimed to nonisothermally study the pyrolysis features and degradation kinetics of the TSPU waste and WPU coating via multi-heating-rate TGA. The investigation of kinetic triplet parameters to mathematically depict the pyrolysis thermal processes of two polyurethane samples is highly important for deeply understanding their degradation processes. Model-free and model-fitting methods can be appropriately carried out for the kinetic analysis purpose, as highly recommended by the ICTAC Kinetics Committee [22]. Model-free methods involve no assumption of any reaction model and thus are able to accurately estimate activation energy *E_k_* as a function of mass conversion degree, and the most frequently applied model-free methods include the integral Starink (SK) [23], Coats–Redfern (CR) [24] and Vyazovkin–Dollimore (VD) [25] methods, and they are used present work to calculate the distribution of activation energy over the entire mass conversion range. In the case of using a model-fitting method, different reaction models are attempted to fit against TGA data and simultaneously ascertain the reaction model and pre-exponential factor. It may be noticed that there are many reaction models that are usually used, identified as n-order chemical reaction models, diffusion-controlling models, phase boundary reaction models, and nucleation and nuclei growth reaction models [26]. Among these models, the n-order reaction model is the simplest and most commonly explored and considered with the aid of the isoconversional Coats–Redfern method. The results of the present work will offer experimental and theoretical information which is necessary and helpful for pyrolytically disposing of PU wastes.

## 2. Materials and Analysis Methods

### 2.1. Materials

The tennis string polyurethane (TSPU) waste samples were collected locally in Tianjin, China, and were made from the same brand ^®^Taan of Shenzhen Taiang Industrial CO., Ltd., Shenzhen, China. The samples, after washing and drying, were cut short and ground by using a mill, resulting in particles with a diameter of <100 um. For comparison purposes, a waterborne polyurethane (WPU) waste sample was also considered, which was made in our laboratory by using isophorone diisocyanate and a polyester polyol [2] and collected from the coating waste of the paper-sizing application. Before experimental measurements, two polyurethane samples after being dried at 120 °C for 20 h were tightly held in a desiccate container.

### 2.2. TG and FTIR Analyses

TGA analysis of two polyurethane samples was performed on a Shimadzu DTG-60 analyzer. For each run, approximately 3~4 mg of the sample was fed into the sample holder and then heated up from ambient to 850 K for TSPU or 800 K for WPU with a purging nitrogen of 30 mL/min. The non-isothermal TGA data were automatically acquired in a non-isothermal mode under a constant heating rate of 5, 10, 15, or 20 K/min.

Fourier transform infrared (FTIR) analysis of TSPU and WPU samples was considered with a wavenumber range of 400~4000 cm^−1^. A Bruker Alpha-H infrared spectrophotometer was conducted with 32 scans for each measurement. The milled sample, after mingled with KBr at a mass ratio of 5:95, was compressed to a disc sample for subsequent FTIR analyses. 

### 2.3. Kinetic Analysis Methods

With non-isothermal mass loss data, pyrolysis kinetics can be analyzed in an isoconversional way so as to understand in-depth the thermal degradation features of various reactants and provide more reliable knowledge required to design any pyrolysis reactor for waste proposal applications.

In general, a pyrolysis process of any solid can be mathematically written as below.

(1) $$\frac{d\alpha }{\mathrm{dt}}=k\left(T\right)f\left(\alpha \right)$$

where *α* is the level of mass conversion given as *α* = (*M*_0_ − *M*_t_)/(*M*_0_ − *M_f_*) where *M*_0_, *M_t_* and *M_f_* can be directly abstracted from experimental TGA results and defined to be the initial sample mass, the sample mass at time *t*, and the final sample mass, respectively. It may be noticed that d*a*/dt is the rate of the sample mass conversion when it rises from 0 to 1. In Equation (1), k(*T*) is the pyrolysis reaction rate constant universally expressed as the Arrhenius formula k(*T*) = *A*exp(−*E_k_*/RT) with T, R, *E_k_* and *A* as the Kelvin temperature (K), universal gas constant (8.314 Jmol^−1^ K^−1^), the apparent activation energy (kJ mol^−1^) and pre-exponential factor (min^−1^), respectively. In the meantime, *f*(*α*) is a differential mass conversion function, and it is merely a temperature-dependent reaction model. Table S1 presents some commonly used reaction mechanism models [26] in differential forms of *f*(*α*) and integral forms of *g*(*α*) considered in the present work. 

Under non-isothermal measuring conditions with *β* = dT/dt, Equation (1) can be transformed as:

(2) $$\frac{d\alpha }{\mathrm{dT}}=\left(\frac{A}{\beta }\right)\exp\left(-\frac{E_{k}}{\mathrm{RT}}\right)\cdot f\left(\alpha \right)$$

Alternatively, Equation (3) can be derived with the reaction mechanism in an integral function form, *g*(*α*), as the following:

(3) $$g\left(\alpha \right)\equiv \int_{0}^{\alpha }\frac{d\alpha }{f\left(\alpha \right)}=\frac{A}{\beta }\int_{0}^{T}\exp\left(-E_{k}/\mathrm{RT}\right)\mathrm{dT}=\frac{A}{\beta }L\left(E_{k},T\right)$$

where *L*(*E*_*k*_,T) is a well-known temperature-integral function that has no analytical solution and can be solved numerically or approximated with different mathematical equations. For the kinetic analysis of various reaction processes, many model-free or model-fitting methods can be attempted [22] for determining the kinetic triplet parameters of *E_k_*, *A* and *f*(*α*)/*g*(*α*). 

The model-free method does not require any assumption of a reaction mechanism model and can be readily and isoconversionally adopted for yielding activation energies as a function of mass conversion. Among various model-free methods, the linear SK method and nonlinear VD method have been highly recommended for achieving more accurate estimates of activation energy. The SK method can be written as:

(4) $$\ln\left(\frac{\beta }{T^{1.92}}\right)=\ln\left[\frac{\mathrm{AR}}{g\left(\alpha \right)}{\left(\frac{E_{k}}{R}\right)}^{-0.92}\right]-1.0008\frac{E_{k}}{\mathrm{RT}}-0.312$$

At any given conversion degree *α*, the plot of ln(*β*/*T*^1.92^) ~ 1/T will lead to a fitted straight line with a slope of 1.0008 *E_k_*/*R*. Subsequently, the *E_k_* value at the given *α* can be easily obtained from the resultant slope value. It may be noticed that the *E_k_*, thus calculated, is independent of the pyrolysis reaction mechanism of polyurethane materials. In the meantime, the CR method [24] is a very popular temperature-integral method and is written as the following linear expression.

(5) $$\ln\left(\frac{\beta }{T^{2}}\right)=\ln\left[\frac{\mathrm{AR}}{E_{k}g\left(\alpha \right)}\right]-\frac{E_{k}}{\mathrm{RT}}$$

Like the SK method, plotting ln (*β*/*T*^2^) versus the reciprocal of temperature at a fixed conversion, *α*, turns to yield a straight line, and from its slope, the *E_k_* values can be obtained. Based on Equation (5), the ln *A* value can be calculated if knowing the most appropriate function *g*(*a*) and usually the first-order reaction with *g*(*α*) = −ln(1 − *α*) is attempted for this consideration.

Unlike the SK and CR methods, the VD method is only focused on the determination of *E*_a_ through a nonlinear minimization procedure, with an objection function, SF, defined as the following:

(6) $$\mathrm{SF}=\left|m\left(m-1\right)-\overset{n}{\underset{i\neq j}{\sum }}\overset{n}{\underset{}{\sum }}\frac{L\left(E_{k}{,T}_{i}\right)\beta_{j}}{L\left(E_{k}{,T}_{j}\right)\beta_{i}}\right|$$

where *m* is the number of the heating rate used to conduct non-isothermal TGA measurements. In the present paper, *L*(*E_k_*,T) is accurately approximated with a 4th order polynomial equation proposed by Senum and Yang [27], which can be written as below:

(7) $$\Omega \left(x\right)=\frac{\exp\left(-x\right)\left(x^{4}+18x^{3}+86x^{2}+96x\right)}{x^{2}\left(x^{4}+20x^{3}+120x^{2}+240x+120\right)}$$

where *x* is set to be *E_k_*/RT and *L*(*E_k_*,T) = *E_k_*/*R·*Ω(*x*). For each *α*, the value of SF can be calculated by substituting experimental values of T and *β* into Equation (6), and it reaches the minimum by numerically varying the *E_k_* value. The nonlinear calculations are performed with the aid of Matlab software, giving an estimate of the activation energy to find the dependence of the activation energy on the extent of pyrolysis mass conversion.

As for the pre-exponential factor lnA, Vyazovkin and his coworkers have highly recommended the use of the model-free compensation effect to accurately obtain it [28,29,30]. Such effect indicates the linear relationship existing between *E_k_* and lnA, and it can be mathematically expressed as:

(8) $${\mathrm{lnA}=a+bE}_{k}$$

where a and b are the characteristic compensation parameters. Once both a and b are available, the lnA can be evaluated by substituting the *E_k_* values. To obtain lnA according to Equation (8), Liavitskaya and Vyazovkin [28] have demonstrated a procedure to accurately determine the parameters of a and b, and following such procedure, a differential expression must be employed and derived by transforming Equation (1) into Equation (9):

(9) $$\ln\left[\left(\frac{d\alpha }{\mathrm{dt}}\right)\right]-\ln\left[f\left(\alpha \right)]\right]=\mathrm{lnA}-\frac{E_{k}}{\mathrm{RT}}$$

The left-hand side of Equation (9) can be computed using a model-fitting method and a pair of parameters, lnA and *E_k_*, are then obtained, respectively, for each reaction model *f*(*α*) shown in Table S1, from the intercept and slope of the left-hand side of Equation (9) linearly drawn against the reciprocal of temperature. The respective pairs from several reaction models are then fitted into Equation (8), leading to the characteristic compensation parameters a and b. Thereafter, Equation (8) is used to compute model-freely the lnA values for all *E_k_* values evaluated by a model-free method, and the VD method is considered here. Prof. Vyazovkin has recently [31] highlighted that using all lnA and *E_k_* pairs, correct or not, the IPF approach is highly accurate for both kinetic single-stage and multi-stage pyrolysis processes. These works [28,29,30,31] have demonstrated that for describing multi-stage pyrolysis for TSPU and WPU, multiple heating rate data may satisfy the obtainment of the compensation effect while for a relatively simple single-stage process, a single heating rate method could suffice to achieve the characteristic compensation parameters.

Moreover, the variation in the lnA values could be better interpreted in terms of the activation entropy change (Δ*S*^#^) other than the change in temperature [31]. Based on the transition state theory, an intimidating product, called a pseudo activated complex, is assumed to form during the reaction process, and then Δ*S*^#^ represents the difference between the entropy of the activated complex and the entropy of the reactants. To represent the reaction equilibrium between the activated complex and the reactants, two other thermodynamic parameters of Δ*G*^#^ and Δ*H*^#^ must be used and they are indicating a similar difference in the enthalpies and the free energies, respectively. Accordingly, an increase in Δ*S*^#^ and a decline in Δ*H*^#^ are both favoring the decrease in Δ*G*^#^ and the obtainment of negative Δ*G*^#^, thereby driving the equilibrium towards the formation of the activated complex. These parameters can be calculated by following the equations given below [31,32,33]:

(10) $$A=\frac{{\mathrm{ek}}_{B}T}{h}\exp\left(\frac{\Delta S}{R}\right)$$

Δ*H*^#^ = *E_k_* − RT (11)

Δ*G*^#^ = Δ*H*^#^ − *T*Δ*S*^#^ (12)

where *k_B_* and *h* are, respectively, the Boltzmann constant (1.381 × 10^−23^ J/K), and Plank constant (6.626 × 10^−34^ J/s) while *e* is the Neper number (2.7183).

Apart from *E_k_* and lnA, the reaction mechanism function *f*(*α*) or *g*(*α*) is a third kinetic parameter and usually, the model-fitting approach has been applied for such a purpose. Following this approach, the reaction function can be obtained by forcing various theoretical reaction models in Table S1 to fit against experimental kinetic data and a specific reaction model can be thought of as the most probable mechanism model if resulting in the best fitting results. Frequently, the CR method has been attempted for model-fitting applications, from which the most appropriate reaction model has been determined accordingly [34,35,36].

## 3. Results and Discussion

### 3.1. FTIR Analysis Results

**Figure 1:**
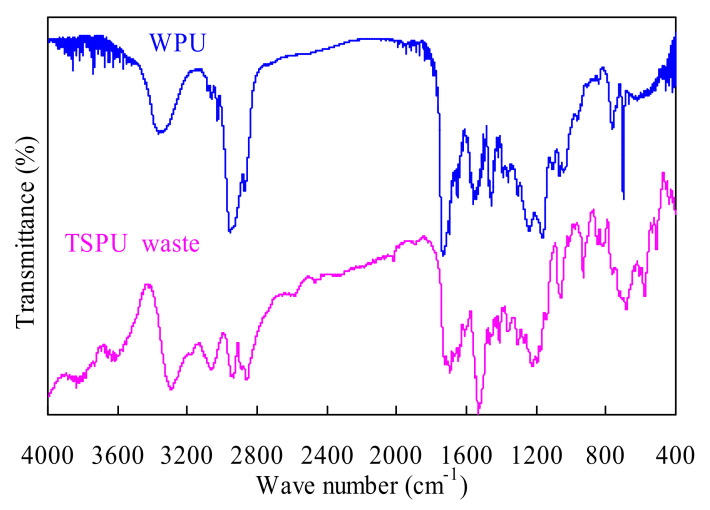
FTIR spectra measured for TSPU waste and WPU samples.

Figure 1 shows the FTIR analysis results for both TSPU and WPU samples, and the FTIR spectra for WPU are almost identical to those reported in the literature [2]. The band peaked at 3365 cm^−^^1^ could be assigned to the stretching vibrations of NH groups while the adsorption bands peaked at 2953, and 2870 cm^−^^1^ might represent the unsymmetrical and symmetrical stretching movements of the CH groups. The sharp peak at 1730 cm^−^^1^ and that at 1558 cm^−^^1^ might, respectively, indicate the presence of C=O and NH groups in the urethane NHCOO groups. In addition, the peak at 1457 cm^−^^1^ could be due to the C=C stretching vibration of the benzene ring and the bands clearly seen at around 762 and 700 cm^−^^1^ could be related to the deformation vibration of the benzene ring. As for the TSPU sample, the band at 3292 cm^−1^ for the NH groups and those at 3065, 2933 and 2854 cm^−1^ for CH_3_ groups can be observed in Figure 1. The bands at 1692 and 1533 cm^−^^1^ could be mainly related to the vibrations of C=O and NH groups in the urethane segments [18,37]. The adsorption band of 1240~1050 cm^−1^ might be assigned to the presence of the C–O–C ether groups.

### 3.2. Thermogravimetric Analyses

#### 3.2.1. Pyrolysis Features at 15 K/min

**Figure 2:**
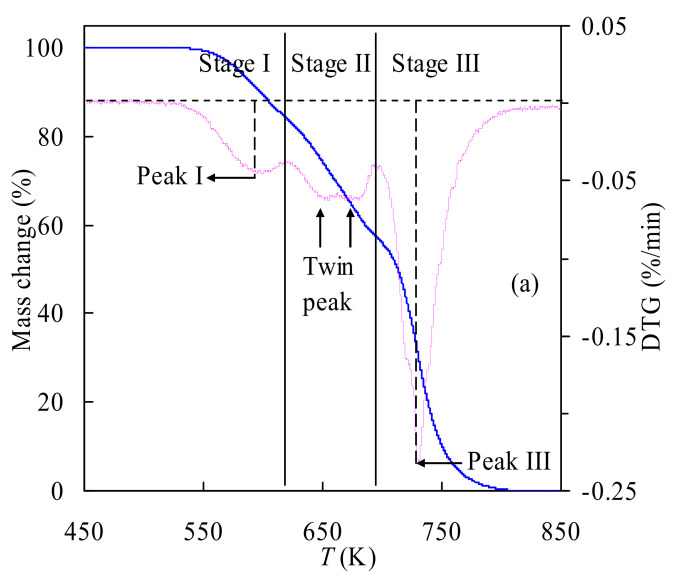

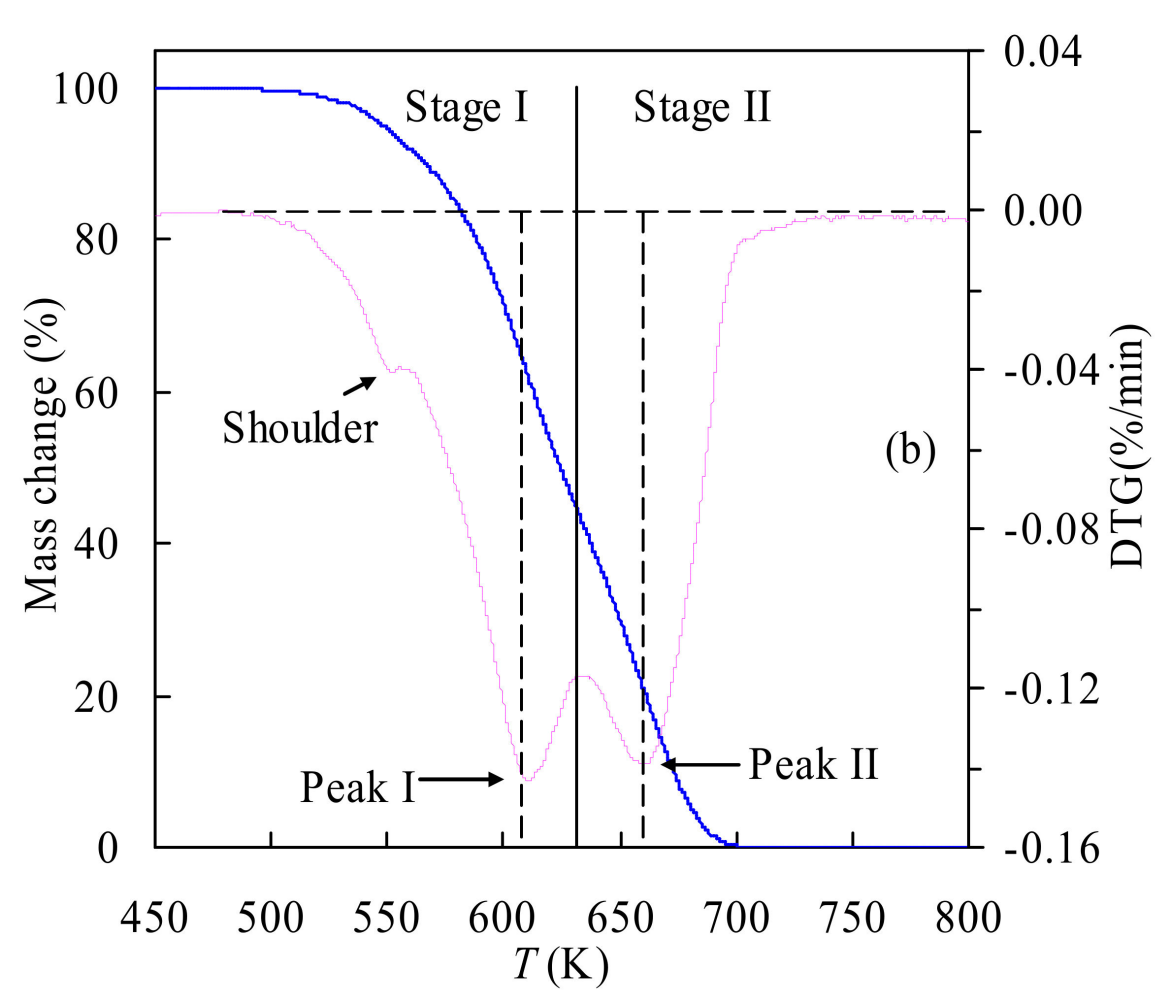
TGA and DTG curves for TSPU (**a**) and WPU (**b**) at *β* = 15 K/min.

Figure 2 shows the temperature-dependent mass variation TGA and mass loss rate DTG curves of two polyurethane samples at 15 K/min in nitrogen. One can observe that for the TSPU sample, its pyrolysis degradation varied in the range of 536–810 K while for the WPU sample, its pyrolysis started at 489 K and ended at 742 K. From the TGA data, it can be deduced that TSPU possesses stronger thermal stability than WPU, possibly arising from its high molecular weight to meet the requirements of high energy tennis sport. On the basis of the DTG results, one can conclude that both polyurethanes are characterized by two or three pyrolysis stages, similar to those addressed earlier. Furthermore, for TSPU, three different stages can be identified upon the variation of the DTG value and they are from 536.2 to 623.1 K, 623.1 to 691.2 K and 691.2 to 810.3 K, respectively; accordingly, their respective mass losses are 16.79%, 24.85% and 58.36%. Among three stages, stage II is composed of twin peaks that are difficult to split from each other. On the other hand, for the WPU sample, two pyrolysis stages can be determined even though they are partially overlapped, and they are 489.2–630.5 and 630.5–742.9 K. It may be noted that there is a shoulder at 550.0 K during pyrolysis stage I. Moreover, the mass losses for Stages I and II are, respectively, 54.76% and 45.24% according to the TGA curve.

#### 3.2.2. Effect of Heating Rate on Pyrolysis Features

**Figure 3:**
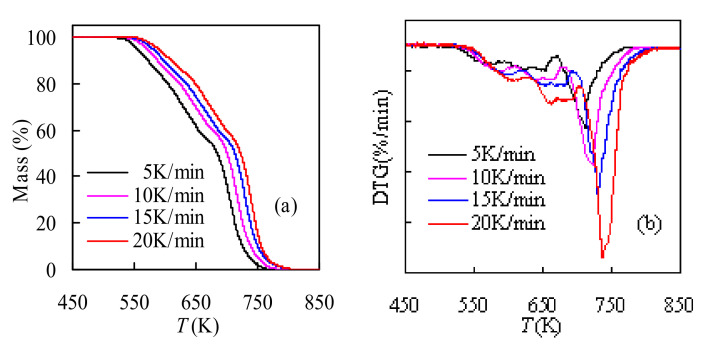

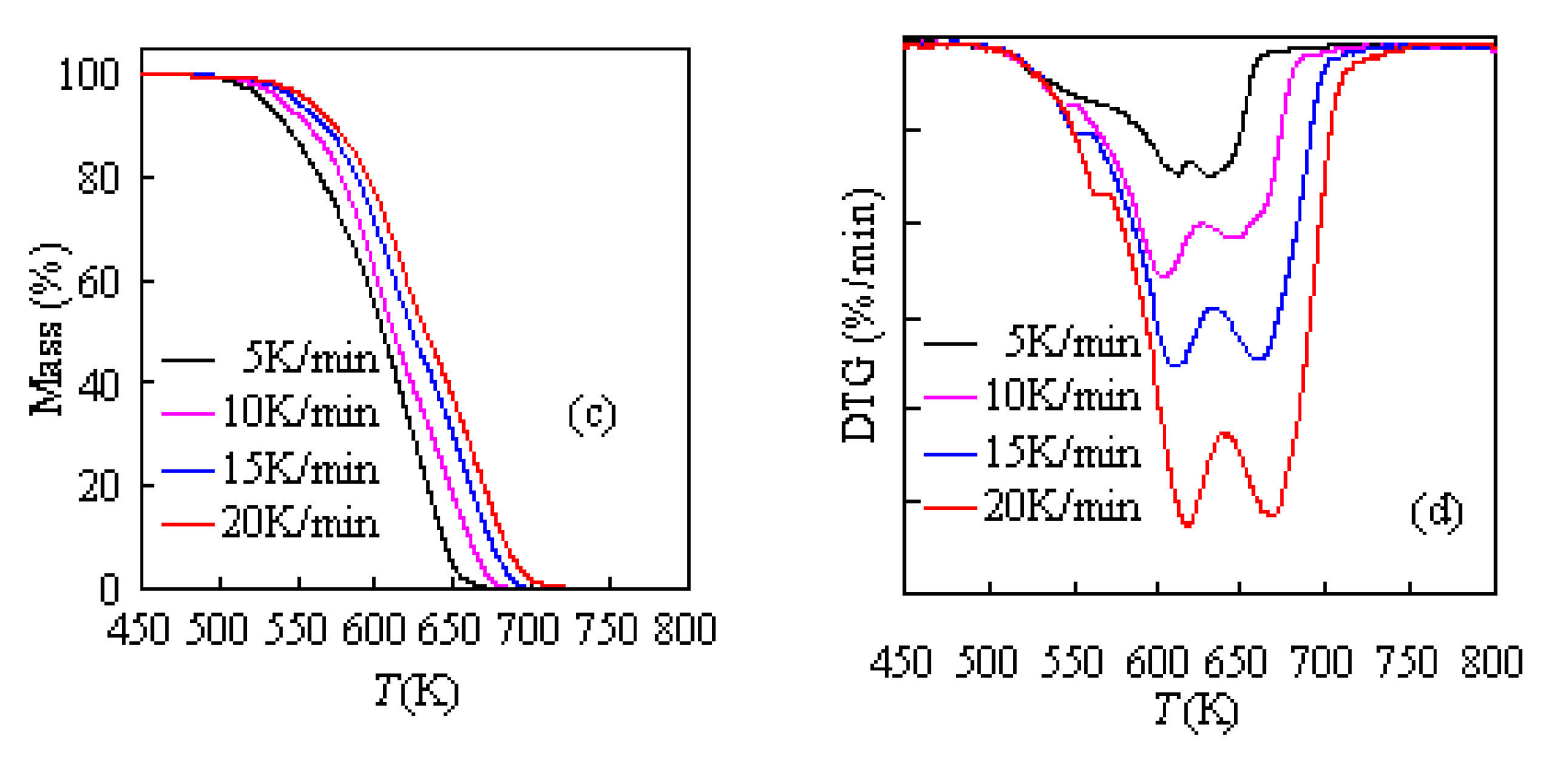
TGA and DTG results determined for TSPU (**a**,**b**) and WPU(**c**,**d**).

Figure 3 describes the impact of the heating rate on the thermal decomposition of TSPU waste and WPU waste samples. It could be noticed in Figure 3 that the shapes of mass loss and DTG curves under the four heating rates are basically similar to each other. The pyrolysis process of TSPU can all be divided into three stages, while for WPU, its pyrolysis can be separated into two stages under different heating rates, and more detailed information is presented in Table 1. However, with an increase in heating rate, the TGA and DTG curves moved to the higher temperature region, as seen in Figure 3, and this behavior is because of the effect of the temperature hysteresis depending on the heating rate. As one knows, heating rates and residence time are inversely competing against each other, and a thermal gradient will have resulted as the residence time will be shortening upon increasing the heating rate. Consequently, the increase in the heat transfer rate between the environment and the sample cannot match the scheduled temperature increase, leading to the decline in the degradation rate and the shifting-to-right of both TGA and DTG curves at high heating rates.

#### 3.2.3. Pyrolysis Performance Indices

For quantitative evaluation of the pyrolysis process, two pyrolysis performance parameters can be attempted for this purpose, and they are the devolatilization index (*DI*) and heat-resistance index (HRI), respectively. These parameters can be calculated using the following expressions [38].

(13) $$\mathrm{HRI}=0.49\times \left[T_{5}+0.6\left(T_{30}-T_{5}\right)\right]$$

(14) $$\mathrm{DI}=\frac{-{\;\mathrm{DTG}}_{\max}}{T_{i}\cdot T_{m}\cdot \Delta T}$$

where *T*_5_ and *T*_30_ are defined as the temperatures at 5% and 30% of mass losses, respectively, and can be extracted from TGA data. DTG_max_ represents the maximum mass loss rate (%/min) over the temperature range of interests, *T_m_* is the temperature at the maximum mass loss rate and ΔT is defined as the temperature range corresponding to the DTG/DTG_max_ = 0.5 and here taken conveniently as the half of the temperature span of interests. *T_i_* is the initial degradation temperature and is simply taken here as the temperature at 1% of the sample mass loss.

For the pyrolysis processes involving multiple stages, the overall DI parameter (DI_c_) for the entire conversion range can be estimated by following the expression given below:

(15) $${\mathrm{DI}}_{o}=\underset{i}{\sum }w_{i}{\mathrm{DI}}_{i}$$

where *w_i_* is the mass loss percentage of pyrolysis stage *i* in the total mass loss (%) and DI*_i_* represents the pyrolysis devolatilization index of stage *i*.

Resultantly, all the pyrolysis characteristic parameters for TSPU and WPU are listed in Tables S2 and S3, respectively. As can be seen, the HRI value rises up as *β* increases, indicative of the increase in the thermal resistance with the heating rate. These findings are consistent with the temperature hysteresis observed in TGA results. In a comparison between TSPU and WPU samples, these HRI values suggest the former is more thermally stable than the latter. The devolatilization index (DI) can be determined by the highest mass loss rate and the duration of pyrolysis degradation and then regarded as a measure to evaluate the pyrolyzablity of the samples of interest. A higher DI value generally indicates easier pyrolysis and better pyrolysis performance. By comparing Table S2 against Table S3, the DI values are seen basically at the same magnitude for both polyurethanes. WPU is observed to demonstrate higher DI than TSPU over the pyrolysis duration, including stage I and stage II, suggesting that WPU is relatively easier to release the volatiles than TSPU. Interestingly, for stage III of TSPU, a much higher DI value is found, indicating that for TSPU, there is a much stronger release of volatiles. Clearly, it is because TSPU has considerably higher DTG_max_ values at stage III, as shown in Table S2.

### 3.3. Kinetic Analysis of Two Polyurethanes

#### 3.3.1. Determination of Activation Energy

##### E_k_ Values from Linear the CR and SK Methods 

**Figure 4:**
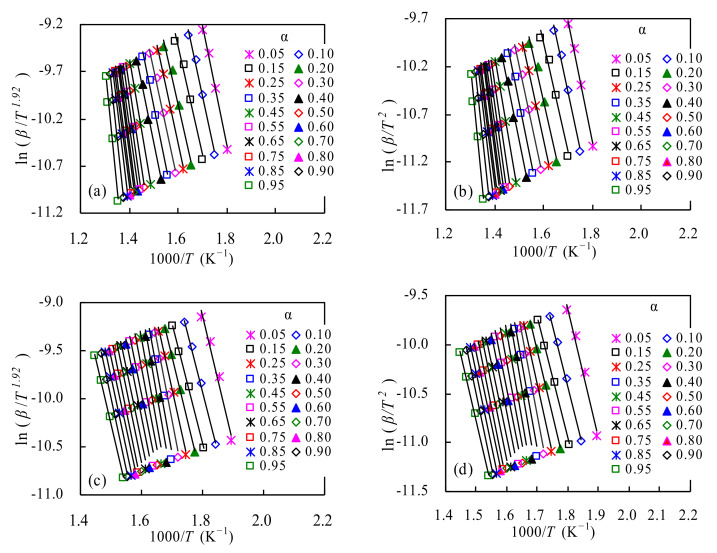
Linear Arrhenius plots for TSPU (**a**,**b**) and WPU (**c**,**d**): (**a**,**c**) SK method ln(*β*/*T*^1.92^) vs. 1000/*T*, and (**b**,**d**) CR method ln(*β*/*T*^2^) vs. 1000/*T*.

Figure 4 presents the Arrhenius plots resulting from both the linear CR and SK methods for TSPU and WPU. By virtue of the ln (*β*/*T*^2^) versus 1000/*T* lines for the CR method and the ln(*β*/*T*^1.92^) vs.1000/*T* lines from the SK method, the a-dependent *E_k_* values can be calculated from the slope of each line and all the calculations are given in Table S4 for TSPU and Table S5 for WPU, respectively. The resultant *R*^2^ values are presented in Tables S4 and S5, as well. As can be seen, these *R*^2^ values for all conversion degrees are very close to 1.00, reflecting a very satisfactory linear relationship for these Arrhenius plots. 

Looking through the two tables, one can see that with the SK method, the *E_k_* values are in the range of 92.84~204.31 kJ/mol for TSPU and 103.64~148.27 kJ/mol for WPU, receptively. If averaged over the whole conversion range, the resultant *E_k_* values are 137.08 and 125.86 kJ/mol for TSPU and WPU, respectively. As such, it may be estimated that the *E_k_* range is 81.3% of the *E_k_* average for TSPU and that is 35.5% for WPU. These relatively large deviations around the averaged *E_k_* value suggest that for both samples, their pyrolysis processes could involve multiple degradation reaction stages and such a reasonable implication is very well consistent with TGA and DTG results. If compared with the literature, the activation energies for TSPU are found to be very similar to those *E_k_* values of 0.1 < α < 0.8 reported by Rosu et al. [14]. In contrast, the averaged *E_k_* values for WPU are much lower than those for WPU [17,18] and its silver composites [18], possibly because their WPU samples have high molecular weights for meeting application requirements [17,18]. 

A careful examination of Tables S4 and S5 indicates that the *α*-dependent *E_k_* results from the CR method for TSPU and WPU are comparable to those from the SK method at each conversion level. The averaged *E_k_* values from the CR method are 136.74 kJ/mol for TSPU and 125.55 kJ/mol for WPU, respectively. Apart from the linear CR and SK methods, the nonlinear VD method [26] has also been attempted with the aid of a minimization program, and the *E_k_* and *SF* values thus determined numerically are also presented in Tables S4 and S5. By this method, the averaged *E_k_* values are 137.15 and 125.91 kJ/mol for TSPU waste and WPU samples, respectively. Among the three isoconversional methods, they all demonstrate a very similar *α*-depending trend for each sample. Furthermore, the *E_k_* values from the SK for all *α* values are almost equal to those of the VD methods, and both methods yield slightly higher *E_k_* values than the CR method. To better describe multi-stage processes, the *E_k_* values obtained with the VD method are also averaged over each stage and given in Table 2 for two PU samples. Unsurprisingly, these results are also different from those reported in the works of literature. For example, Rosu et al. [14] have reported that for four stages, the averaged *E_k_* are, respectively, 120, 139, 183 and 324 kJ/mol [14], rather higher than those obtained for two PU samples, as shown in Table 2.

#### 3.3.2. Determination of the Pre-Exponential Factor lnA

**Figure 5:**
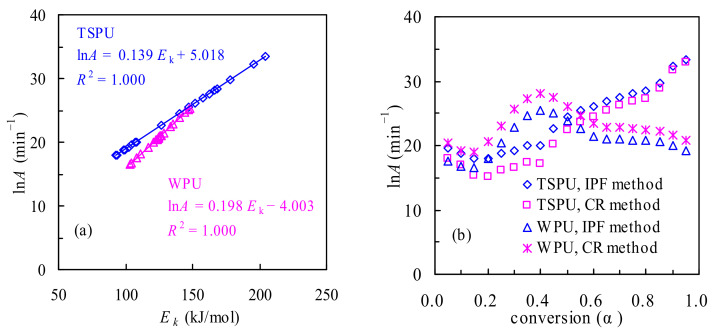
Compensation effect (**a**) and lnA~*α* relationship (**b**) for TSPU and WPU.

For multiple stage pyrolysis processes, the model-free IPF method has been highly recommended to obtain the pre-exponential factor lnA values [22,28,29,30,31]. Following such a method, the linear compensation effect and lnA ~ *α* results can be determined and are graphically shown in Figure 5 for two polyurethane samples. According to the work of Liavitskaya and Vyazovkin [28], four reaction models, including three power-law nucleation models (P1/4, P1/3 and P1/2) and the Avrami–Eroféev random nucleation model (A2), are good enough to accurately yield the compensation effect parameters, and these four models are given in Table S1, as well. Using the aforementioned four models considered, the a and b values are obtained hereby fully considering kinetic data acquired under the four heating rates of 5, 10, 15 and 20 K/min for multi-stage pyrolysis processes of TSPU and WPU. Consequently, by plugging the *E_k_* from the VD method along with a and b into Equation (8), the lnA can be yielded and the lnA values for each pyrolysis stage are given in Table 2 while the lnA ~ *α* dependency results are presented in Figure 5 for TSPU and WPU. Seemingly, the *α*-dependent lnA in Table S6 has followed the variation trend of *E_k_* with *α*.

As can be seen from Table S6, the lnA from the IPF method for the TSPU waste ranges from 17.94 to 33.42, or the A value spans from 6.18 × 10^7^ to 3.26 × 10^14^ min^−1^, correspondently. In the case of the WPU waste, the lnA value is seen to vary from 16.56 to 25.41 or the *A* value ranges from 1.56 × 10^7^ to 1.07 × 10^11^ min^−1^, heavily dependent on the pyrolysis mass conversion level. On average, the lnA values thus obtained are 24.08 and 20.96 for TSPU and WPU, respectively. Shown in Figure 5b, also includes the *α*-dependent lnA results over the range of 0.05 < *α* < 0.95 from the CR method, where the first-order reaction assumption and *E_k_* values are taken. As shown in Figure 5b, the lnA values from the CR and IPF methods have demonstrated almost the same trend for each polyurethane sample, but the lnA differences between the two methods are relatively considerable. As clearly seen, the lnA values from the IPF method are overall higher than those by using the CR method for the TSPU waste, whereas the opposite finding is applied for the WPU waste. In the meantime, lnA can be obtained like the *E_k_* for three stages of TSPU and two stages of WPU samples, and the averaged lnA values are also presented in Table 2.

Once the *E_k_* and lnA are available, the parameters of Δ*G*^#^, Δ*H*^#^ and Δ*S*^#^ can be calculated using Equations (10)–(12), and here, the *E_k_* values used were those from the VD method. The results thus obtained are presented in Table S6. As seen, all the Δ*S*^#^ values for WPU and most Δ*S*^#^ ones for TSPU are negative, indicating that the degrees of freedom for the activated complex are diminishing relative to the initial reactants. It is not out of expectation since the activated complex is thermodynamically more structure-ordered than the reactant of the system and negative Δ*S*^#^ indicates the decelerated process. Interestingly, the Δ*S*^#^ values are seen to follow the same trend of the change in lnA and thereby the relationship between Δ*S*^#^ and lnA is barely free of temperature. 

The enthalpy change (Δ*H*) is a parameter to discriminate the energy between the reaction system and the surrounding, externally appearing as either an endothermic or exothermic process. It can be seen that the Δ*H*^#^ are varying between 87.89~198.04 kJ/mol for TSPU and 98.96~143.25 kJ/mol for WPU, respectively. These results may suggest that pyrolysis reactions of two waste samples are both heat-absorbed, thus requiring more energy to thermally crack polymer bonds into low molecular weight compounds. Clearly, it is true for achieving pyrolysis of TSPU and WPU by external heating. In the meantime, Δ*G*^#^ is a parameter to quantify the change in free energy between the reaction system and the surrounding, as a combined result of Δ*H*^#^ and Δ*S*^#^. As well, the Δ*G*^#^ values are seen to range from 154.94 to 185.30 kJ/mol over 0.05 < α < 0.95 for TSPU, and narrows a little down from 165.04 to178.75 kJ/mol for WPU, respectively. From a thermodynamic point of view, the positive ΔG suggests that the pyrolysis for two samples is not a spontaneous process. The lower the ΔG the more favorable the pyrolysis to occur, and the less energy is required accordingly.

#### 3.3.3. Determination of the Most Probable Reaction Model

**Figure 6:**
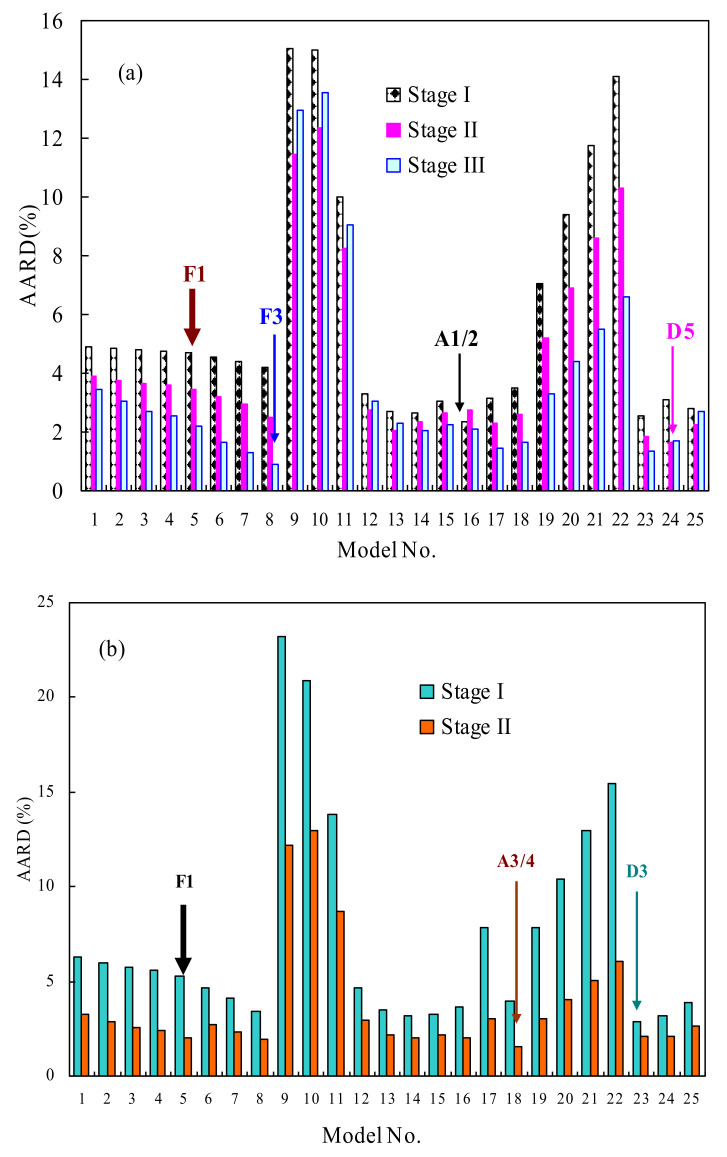
The AARD results for some reaction models along with the CR method for multi-stage pyrolysis processes of TSPU (**a**) and WPU (**b**).

After obtaining the *E_k_* and lnA, the third kinetic parameter of the pyrolysis reaction mechanism model is also identified by using the model-fitting method for pyrolysis of TSPU waste and WPU counterpart. For this purpose, the Coats–Redfern methodology has been used, along with 25 reaction models given in Table S1, by assuming one global reaction model for the entire kinetic degradation process or individually simulating each pyrolysis stage process with these models. The fitting performance evaluation for all reaction models has been done by using the overall absolute average relative deviation (*AARD*) defined as $\mathrm{AARD}\%=\sum \left|1-\alpha_{\mathrm{cal}}/\alpha_{\exp}\right|/N\times 100$, where *a_cal_* and *α*_exp_ are, respectively, the calculated conversion values and experimental data while *N* is the total number of all the *α*-*T* data points over four heating rates. Therefore, it may be thought that the smaller the AARD value is, the more appropriate to describe the pyrolysis the reaction model considered, and then the most suitable model *g*(*α*) may be determined if its AARD value is the lowest among all the models. Figure 6 presents the AARD calculations with 25 reaction models for multi-stage pyrolysis processes in TSPU and WPU samples. It can be seen from Figure 6a that the A1/2, D5 and F3 models have led to the lowest AARD value for Stages I, II and III of TSPU pyrolysis, respectively. As well, the D3 and A3/4 models are, respectively, found to yield the lowest AARD value for Stages I and II of WPU pyrolysis, as reflected by Figure 6b. In the meantime, if simply using the one global reaction assumption, the F3 and D3 models are thought to be the most probable reaction model *g*(*α*), as shown in Figure S1 for describing pyrolysis of TSPU and WPU wastes, respectively.

**Figure 7:**
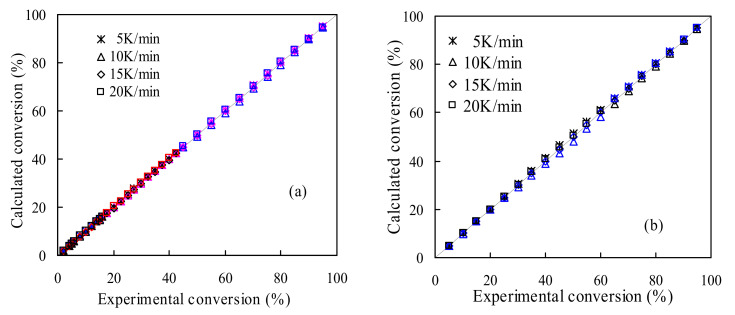
Simulated results for multi-stage pyrolysis processes of TSPU (**a**) and WPU (**b**).

Figure 7 has graphically presented the matching results versus experimental values with the A1/2, D5 and F3 models for TSPU and the D3 and A3/4 models for WPU, respectively. It can be observed that these models have performed excellently for two PU wastes and all data points have almost coincided on the diagonal line. On the other hand, the global reaction models of F3 and D3 have also achieved satisfactory fittings for respective pyrolysis processes of TSPU and WPU, although certain deviations from the diagonal line can be observed in Figure S2.

In addition, the F1 reaction model has also been examined because this model has been reported to satisfactorily depict kinetic pyrolysis of polyurethane [10,18] and it has led to relatively lower AARD values for both TSPU and WPU than the other models, as shown in Figure 6. In the meantime, the comparison between experimental α data and calculated values has been made and graphically presented in Figure S2. As can be seen, the F1 model has resulted in fairly good simulations against experimental results but caused relatively larger scatterings along the diagonal line for two polyurethanes than their respective best reaction models.

## 4. Conclusions

With the present work, pyrolysis processes of TSPU waste and WPU waste have been experimentally studied by means of multi-heating-rate TGA measurements. A detailed comparison of TSPU and WPU wastes has been made in terms of pyrolysis features and pyrolysis performance indices. For kinetic analysis of non-isothermal data, linear SK and CR methods, and nonlinear VD methods were attempted to calculate activation energies over the entire conversion range. With the multi-stage IPA and model-fitting CR methods, the lnA values and the pyrolysis degradation model have been obtained for pyrolysis of TSPU and WPU wastes. Some remarks may be drawn as the following:

(1) The non-isothermal TGA-DTG results suggest that TSPU waste has higher thermal stability than WPU, possibly related to the difference in their macromolecular weights. They are both characteristic of multiple pyrolysis processes, mainly completed in three stages for TSPU and two stages for WPU.
(2) Pyrolysis characteristics can be better interpreted in terms of the heat-resistance index (HRI) and devolatilization index (DI). The HRI value increases with the heating rate and is consistent with the temperature hysteresis observed, and TSPU appears to have higher HRI values than WPU, indicative of its more pyrolysis stability. Furthermore, WPU seems to have higher DI values than TSPU, suggesting it is easier pyrolysis and better pyrolysis performance.
(3) Kinetic analysis was done for pyrolysis of TSPU and WPU by using the model-free SK, CR and VD methods, successfully achieving the α-dependent *E_k_*. The averaged *E_k_* values thus obtained with the three methods are 137.08, 136.74 and 137.15 kJ/mol for TSPU, 125.86, 125.55 and 125.91 kJ/mol for WPU, respectively. These results further suggest TSPU is thermally more stable than WPU, thus requiring more energy for pyrolysis to happen.
(4) For multi-stage pyrolysis of two wastes, the isoconversional lnA values were calculated by using the model-free IPA method, and the averaged lnA values are 24.08 and 20.96 min^−1^, respectively, for TSPU and WPU. Three thermodynamic parameters of Δ*G*^#^, Δ*H*^#^ and Δ*S*^#^ were estimated as well, and negative Δ*S*^#^, positive Δ*G*^#^ and Δ*H*^#^ were obtained for two samples, reflecting that their pyrolysis is characteristic of a deceleration process of losing free degree, and it was thermodynamically less favorable but absorbing heat.
(5) By using the model-fitting with the aid of the CR method, the simulation performances of 25 models were scanned and the A1/2, D5 and F3 models were found to be the most appropriate to represent the three-stage pyrolysis processes for TSPU, and the D3 and A3/4 models for two-stage pyrolysis degradations of WPU, resulting in very satisfactory simulation performances against experimental data.
(6) The present work has provided prerequisite information about pyrolysis features and kinetic triplet parameters of two PU waste samples necessary for subsequent pyrolysis reactor design for the purpose of thermal disposal of PU wastes. However, the PU wastes in reality are mostly mixed with other wastes, such as biomass, plastics or metal-containing species, and thus further work on mixed PU wastes should be carried out so as to provide solid support for better thermal treatment of real wastes.

## Figures and Tables

**Table 1:** Temperature ranges for multi-stage pyrolysis of two polyurethane samples.

Items	*β* (K/min)			
	5	10	15	20
TSPU				
Stage I (K)	524.8–594.4	530.1–608.1	536.2–623.1	545.2–630.6
*w*_I_ (%)	16.72	15.67	16.79	15.00
Stage II (K)	594.4–671.4	608.1–677.5	623.1–691.2	630.6–705.7
*w*_II_ (%)	27.86	24.69	24.85	26.98
Stage III (K)	671.4–769.4	677.5–785.0	691.2–810.3	705.7–809.4
*w*_III_ (%)	55.42	59.64	58.36	58.02
WPU				
Stage I	467.2–617.6	480.1–624.0	489.2–630.5	491.7–639.6
*w*_I_ (%)	62.90	60.14	54.76	55.17
Stage II	617.6–717.0	624.0–739.3	630.5–742.9	639.6–748.8
*w*_II_ (%)	37.097	39.86	45.24	44.83

**Table 2:** Kinetic parameters characterizing the pyrolysis stages of PU *.

Samples	Parameters	Stage I	Stage II	Stage III
TSPU	*E_k_* (kJ/mol)	98.98	102.21	163.45
	lnA (min^−1^)	18.78	19.22	27.74
WPU	*E_k_* (kJ/mol)	127.26	124.05	
	lnA (min^−1^)	21.23	20.59	

* *E_k_* from VD method and lnA from IPF method.

## Data Availability

Here, we declare that the data presented in this study are available on request from the corresponding author.

## Supplementary Materials

The following supporting information can be downloaded at: https://www.mdpi.com/article/10.3390/polym14081501/s1, Table S1: Some reaction models of g(α) and f(α) for describing solid-state reactions; Table S2: Pyrolysis characteristic parameters for TSPU under different heating rates; Table S3: Pyrolysis characteristic parameters for WPU under different heating rates; Table S4: Activation energies estimated for pyrolysis of TSPU waste; Table S5: Activation energies estimated for pyrolysis of WPU waste; Table S6: Thermodynamic parameters estimated for pyrolysis of two polyurethanes; Figure S1: The AARD results for some reaction models via the CR method with the one global reaction model assumption for the entire pyrolysis of two PU samples; Figure S2: Simulated results obtained with the one global reaction model for pyrolysis processes of TSPU (a,b) and WPU (c,d)
