# Supplementary material for: Pyrolysis Evaluation of Tennis String Polyurethane and Water-Borne Polyurethane Wastes through Isoconversional Kinetic Analysis
Source: Polymers (Basel). 2022 Apr 7;14(8):1501. doi: 10.3390/polym14081501 (PMC9025778; doi:10.3390/polym14081501)
Supplement: Supplementary file 1 [file polymers-14-01501-s001.zip › polymers-1610833-supplementary.pdf]

Supplementary Materials

Pyrolysis Evaluation of Tennis String Polyurethane and Water-Borne Polyurethane Wastes through Isoconversional Kinetic Analysis

Hai-bo Wan [1] and Zhen Huang [2,*]

[1] Department of Physical Education, Tianjin University of Commerce, Tianjin 300134, China;
wanhaibo@tjcu.edu.cn
[2] Department of Packaging Engineering, Tianjin University of Commerce, Tianjin 300134, China
* Correspondence: huangzhen@tjcu.edu.cn; Tel.: +86-22-26686264; Fax: +86-22-26686251

Table S1 Some reaction models of $g(\alpha)$ and $f(\alpha)$ for describing solid-state reactions

No.	Model	$g(\alpha)$	$f(\alpha)$
Chemical reaction mechanism			
1	F1/3	$1-(1-\alpha)^{2/3}$	$(3/2)(1-\alpha)^{1/3}$
2	F1/2	$1-(1-\alpha)^{1/2}$	$2(1-\alpha)^{1/2}$
3	F2/3	$1-(1-\alpha)^{1/3}$	$3(1-\alpha)^{2/3}$
4	F3/4	$1-(1-\alpha)^{1/4}$	$4(1-\alpha)^{3/4}$
5	F1	$-\ln(1-\alpha)$	$1-\alpha$
6	F3/2	$(1-\alpha)^{-1/2}-1$	$2(1-\alpha)^{3/2}$
7	F2	$(1-\alpha)^{-1}-1$	$(1-\alpha)^{2}$
8	F3	$(1-\alpha)^{-2}-1$	$(1/2)(1-\alpha)^{3}$
Power law nucleation mechanism			
9	P1/4	$\alpha^{1/4}$	$4\alpha^{3/4}$
10	P1/3	$\alpha^{1/3}$	$3\alpha^{2/3}$
11	P1/2	$\alpha^{1/2}$	$2\alpha^{1/2}$
12	P3/2	$\alpha^{3/2}$	$(2/3)\alpha^{-1/2}$
13	P2	α^{2}	$(1/2)\alpha^{-1}$
Avrami−Eroféev random nucleation mechanism			
14	A1/4	$[-\ln(1-\alpha)]^{4}$	$1/4(1-\alpha)[-\ln(1-\alpha)]^{-3}$
15	A1/3	$[-\ln(1-\alpha)]^{3}$	$1/3(1-\alpha)[-\ln(1-\alpha)]^{-2}$
16	A1/2	$[-\ln(1-\alpha)]^{2}$	$1/2(1-\alpha)[-\ln(1-\alpha)]^{-1}$
17	A2/3	$[-\ln(1-\alpha)]^{3/2}$	$(2/3)(1-\alpha)[-\ln(1-\alpha)]^{-1/2}$
18	A3/4	$[-\ln(1-\alpha)]^{4/3}$	$(3/4)(1-\alpha)[-\ln(1-\alpha)]^{-1/3}$
19	A3/2	$[-\ln(1-\alpha)]^{2/3}$	$(3/2)(1-\alpha)[-\ln(1-\alpha)]^{1/3}$
20	A2	$[-\ln(1-\alpha)]^{1/2}$	$2(1-\alpha)[-\ln(1-\alpha)]^{1/2}$
21	A5/2	$[-\ln(1-\alpha)]^{2/5}$	$(5/2)(1-\alpha)[-\ln(1-\alpha)]^{3/5}$
22	A3	$[-\ln(1-\alpha)]^{1/3}$	$3(1-\alpha)[-\ln(1-\alpha)]^{2/3}$
Diffusion controlling mechanism			
23	D3	$[1-(1-\alpha)^{1/3}]^{2}$	$(3/2)(1-\alpha)^{2/3}[1-(1-\alpha)^{1/3}]^{-1}$
24	D5	$[(1-\alpha)^{-1/3}-1]^{2}$	$(3/2)(1-\alpha)^{4/3}[(1-\alpha)^{-1/3}-1]^{-1}$
25	D6	$[(1+\alpha)^{1/3}-1]^{2}$	$(3/2)(1+\alpha)^{2/3}[(1+\alpha)^{1/3}-1]^{-1}$

Table S2 Pyrolysis characteristic parameters for TSPU under different heating rates

Items	β (K/min)			
	5	10	15	20
T_i (K)	534.2	548.4	556.0	562.9
T_5 (K)	555.0	569.9	579.5	588.4
T_{30} (K)	629.8	649.5	660.8	674.0
HRI (K)	293.9	302.7	307.8	313.5
T_{m1} (K)	571.9	587.7	598.2	602.8
DTG_{max1}(%/min)	−0.037	−0.041	−0.050	−0.021
ΔT_1(K)	34.6	39.0	43.5	42.7
DI_1($\times 10^9 \cdot K^{-3}$)	3.46	3.23	3.44	1.45
T_{m2}	653.9	641.0	679.5	662.6
DTG_{max3}(%/min)	−0.048	−0.054	−0.068	−0.057
ΔT_2(K)	38.5	34.7	34.1	37.6
DI_2($\times 10^9 \cdot K^{-3}$)	3.57	4.41	5.29	4.09
T_{m3}	712.1	722.2	730.5	736.4
DTG_{max3}(%/min)	−0.154	−0.176	−0.234	−0.321
ΔT_3(K)	49.0	53.8	59.6	51.9
DI_3($\times 10^9 \cdot K^{-3}$)	8.24	8.26	9.67	14.95
DI_o($\times 10^9 \cdot K^{-3}$)	6.14	6.52	7.54	10.00

Table S3 Pyrolysis characteristic parameters for WPU under different heating rates

Items	β (K/min)			
	5	10	15	20
T_i (K)	506.1	510.3	519.4	522.8
T_5 (K)	527.6	539.2	548.2	556.6
T_{30} (K)	581.7	591.8	602.0	609.5
HRI (K)	274.4	279.7	284.4	288.3
T_{m1} (K)	611.2	601.5	609.3	616.4
DTG_{max1}(%/min)	−0.057	−0.105	−0.145	−0.244
ΔT_1(K)	75.2	72.0	70.7	74.0
DI_1($\times10^9\cdot K^{-3}$)	2.46	4.75	6.48	10.25
T_{m2}	631.0	646.3	658.9	667.1
DTG_{max2}(%/min)	−0.057	−0.088	−0.141	−0.239
ΔT_2(K)	49.7	57.7	56.2	54.6
DI_2($\times10^9\cdot K^{-3}$)	3.58	4.65	7.34	12.53
DI_o($\times10^9\cdot K^{-3}$)	2.88	4.71	6.87	11.27

Table S4 Activation energies estimated for pyrolysis of TSPU waste

α	SK method		CR method		VD method	
	E_k(kJ/mol)	R^2	E_k(kJ/mol)	R^2	E_k(kJ/mol)	*SF*
0.05	104.66	0.9959	104.37	0.9958	104.73	0.0151
0.10	99.16	0.9875	98.85	0.9874	99.26	0.0452
0.15	92.84	0.9775	92.51	0.9773	92.96	0.0803
0.20	93.57	0.9854	93.23	0.9853	93.70	0.0518
0.25	98.88	0.9905	98.53	0.9904	99.00	0.0339
0.30	102.37	0.9914	102.02	0.9914	102.50	0.0306
0.35	107.44	0.9919	107.08	0.9918	107.56	0.0291
0.40	108.15	0.9910	107.79	0.9909	108.28	0.0325
0.45	126.32	0.9969	125.96	0.9969	126.41	0.0115
0.50	140.03	0.9943	139.67	0.9942	140.09	0.0214
0.55	147.11	0.9869	146.75	0.9868	147.16	0.0493
0.60	151.99	0.9848	151.63	0.9847	152.03	0.0573
0.65	157.49	0.9826	157.14	0.9825	157.54	0.0658
0.70	162.11	0.9819	161.76	0.9817	162.14	0.0689
0.75	166.01	0.9809	165.66	0.9808	166.05	0.0725
0.80	168.51	0.9836	168.16	0.9835	168.54	0.0623
0.85	177.91	0.9874	177.56	0.9874	177.93	0.0481
0.90	195.71	0.9945	195.37	0.9945	195.72	0.0212
0.95	204.31	0.9889	203.98	0.9888	204.31	0.0432
average	137.08		136.74		137.15	

Table S5 Activation energies estimated for pyrolysis of WPU waste

α	SK method		CR method		VD method	
	E_k(kJ/mol)	R^2	E_k(kJ/mol)	R^2	E_k(kJ/mol)	*SF*
0.05	108.67	0.9869	108.40	0.9868	108.72	0.0490
0.10	104.18	0.9988	103.89	0.9988	104.25	0.0043
0.15	103.64	0.9995	103.34	0.9995	103.71	0.0018
0.20	111.28	0.9989	110.98	0.9988	111.34	0.0042
0.25	122.64	0.9920	122.35	0.9919	122.69	0.0301
0.30	135.46	0.9788	135.17	0.9787	135.48	0.0807
0.35	144.58	0.9565	144.29	0.9563	144.60	0.1674
0.40	148.27	0.9365	147.99	0.9361	148.29	0.2461
0.45	146.63	0.9182	146.33	0.9177	146.64	0.3175
0.50	140.24	0.9176	139.94	0.9172	140.26	0.3176
0.55	133.86	0.9285	133.56	0.928	133.90	0.2732
0.60	128.34	0.9440	127.98	0.9436	128.39	0.2117
0.65	126.03	0.9617	125.71	0.9614	126.08	0.1438
0.70	126.35	0.9727	126.03	0.9725	126.41	0.1021
0.75	125.15	0.9829	124.82	0.9827	125.21	0.0637
0.80	124.58	0.9892	124.24	0.9891	124.64	0.0402
0.85	123.53	0.9944	123.19	0.9943	123.61	0.0207
0.90	121.08	0.9968	120.74	0.9968	121.16	0.0118
0.95	116.83	0.9889	116.48	0.9983	116.92	0.0062
average	125.86		125.55		125.91	

Table S6 Thermodynamic parameters estimated for pyrolysis of two polyurethanes

α	TSPU				WPU			
	ln$A$ (min^{-1})	$\Delta H^{\#}$ (kJ/mol)	$\Delta S^{\#}$ (J/mol K)	$\Delta G^{\#}$ (kJ/mol)	ln$A$ (min^{-1})	$\Delta H^{\#}$ (kJ/mol)	$\Delta S^{\#}$ (J/mol K)	$\Delta G^{\#}$ (kJ/mol)
0.05	19.58	99.96	−95.91	154.94	17.55	104.22	−112.26	165.04
0.10	18.82	94.34	−102.49	154.98	16.67	99.60	−119.88	166.61
0.15	17.94	87.89	−110.03	155.01	16.56	98.96	−120.95	168.04
0.20	18.04	88.48	−109.41	157.17	18.07	106.51	−108.51	169.50
0.25	18.78	93.67	−103.46	160.01	20.32	117.80	−89.91	170.69
0.30	19.27	97.07	−99.58	162.14	22.86	130.53	−68.92	171.54
0.35	19.97	102.02	−93.89	164.56	24.67	139.61	−53.97	172.03
0.40	20.07	102.64	−93.21	165.92	25.40	143.25	−47.96	172.33
0.45	22.59	120.63	−72.46	171.03	25.07	141.55	−50.75	172.60
0.50	24.49	134.23	−56.77	174.27	23.81	135.13	−61.35	173.00
0.55	25.47	141.25	−48.67	175.86	22.55	128.72	−71.91	173.53
0.60	26.15	146.08	−43.09	176.93	21.45	123.16	−81.07	174.17
0.65	26.92	151.56	−36.77	178.02	21.00	120.80	−84.96	174.77
0.70	27.56	156.13	−31.50	178.91	21.06	121.08	−84.49	175.24
0.75	28.10	160.01	−27.02	179.65	20.82	119.83	−86.54	175.79
0.80	28.45	162.46	−24.19	180.15	20.71	119.22	−87.55	176.29
0.85	29.75	171.81	−13.39	181.67	20.51	118.14	−89.32	176.86
0.90	32.22	189.54	7.09	184.28	20.02	115.65	−93.43	177.62
0.95	33.42	198.04	16.89	185.30	19.18	111.34	−100.51	178.75

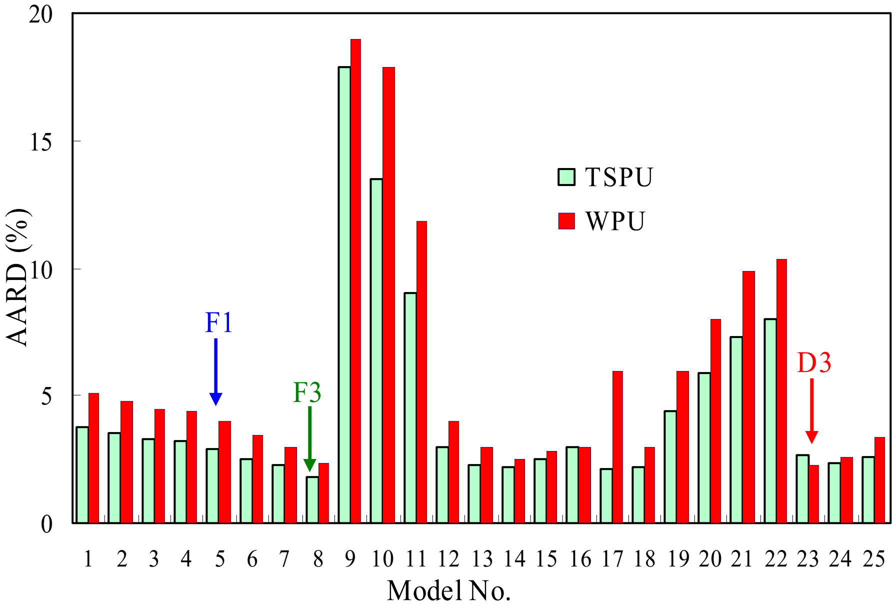

Figure S1 The *AARD* results for some reaction models via the CR method with the one global reaction model assumption for the entire pyrolysis of two PU samples

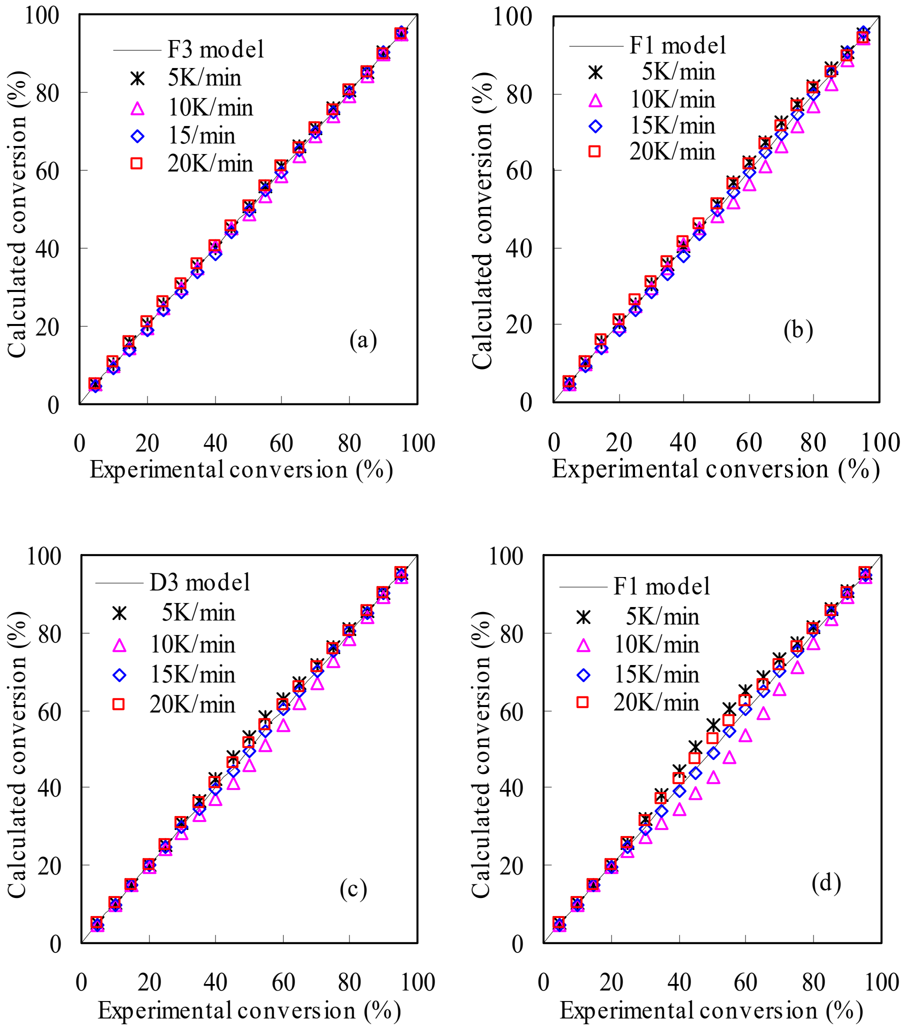

Figure S2 Simulated results obtained with the one global reaction model for pyrolysis processes of TSPU (a, b) and WPU (c, d)
